# DyVarMap: Integrating Conformational Dynamics and Interpretable Machine Learning for Cancer-Associated Missense Variant Classification in FGFR2

**DOI:** 10.3390/bioengineering13010126

**Published:** 2026-01-22

**Authors:** Yiyang Lian, Amarda Shehu

**Affiliations:** 1 School of Systems Biology, George Mason University, Manassas, VA 20110, USA; ylian@gmu.edu; 2 Department of Computer Science, George Mason University, Fairfax, VA 22030, USA

**Keywords:** FGFR2, missense variant, conformational dynamics, AlphaFold2, machine learning, variant effect prediction, precision oncology, receptor tyrosine kinase

## Abstract

Accurate interpretation of missense variants in cancer-associated genes remains a critical challenge in precision oncology, as most sequence-based predictors lack mechanistic explanations. Receptor tyrosine kinases like FGFR2 exemplify this problem: their function depends on conformational dynamics, yet most variants remain classified as variants of uncertain significance (VUS). In this paper we present DyVarMap, an interpretable structural-learning framework that integrates AlphaFold2-based ensemble generation with physics-driven refinement, manifold learning, and supervised classification using five biophysically motivated geometric features. Applied to FGFR2, the framework generates diverse conformational ensembles, identifies metastable states through nonlinear dimensionality reduction, and classifies pathogenicity while providing mechanistic attributions via SHAP analysis. External validation on ten kinase-domain variants yields an AUROC of 0.77 with superior calibration (Brier score = 0.108) compared to PolyPhen-2 (0.125) and AlphaMissense (0.132). Feature importance analysis consistently identifies K659–E565 salt-bridge distance and DFG motif dihedral angles as top predictors, directly linking predictions to known activation mechanisms. Case studies of borderline variants (A628T, E608K, L618F) demonstrate the framework’s ability to provide structurally coherent mechanistic explanations. DyVarMap bridges the gap between static structure prediction and dynamics-aware functional assessment, generating testable hypotheses for experimental validation and demonstrating the value of incorporating conformational dynamics into variant effect prediction for precision oncology.

## 1. Introduction

Advances in next-generation sequencing have uncovered thousands of missense variants in oncogenic kinases [1], yet most remain classified as variants of uncertain significance (VUS) because the molecular mechanisms by which they alter activity are poorly understood [2]. Deciphering these mechanisms is critical: kinases are among the most druggable classes of cancer drivers, and accurate variant interpretation can guide precision therapies [3]. In FGFR2 alone, over 200 distinct missense variants have been cataloged across cancer types, including cholangiocarcinoma, endometrial carcinoma, and bladder cancer, yet fewer than 30% have definitive functional characterization [4].

The functional impact of a missense variant is encoded in the three-dimensional architecture and dynamics of its protein. Those same structural details guide structure-based drug discovery. Therefore, having accurate models, now widely available through deep learning-based predictors such as AlphaFold2, is indispensable for variant interpretation and therapeutic design [5]. AlphaFold2 and related methods now generate atomic protein models at the proteome scale [6], but they provide only static structures [7,8,9]. Kinase regulation, by contrast, is governed by conformational transitions—activation-loop rearrangements, lysine–glutamate salt-bridge toggling, and DFG flips—that occur on micro- to millisecond timescales [10,11,12,13,14]. Ignoring these dynamics can obscure how a single amino-acid change shifts the free-energy landscape toward inactive or overactive states [15,16,17].

Physics-based molecular dynamics (MD) can capture these motions, but hundreds of microseconds of simulation per variant remain impractical for large-scale screening [18]. Likewise, sequence-only variant-effect predictors (e.g., PolyPhen-2 [19], AlphaMissense [20]) are fast but often miss mutations whose impact is structural rather than evolutionary [21]. What is needed is a middle-ground method that retains the speed of AI-enabled structure prediction while incorporating sufficient physics to expose functionally relevant conformations—that is, we need to bridge static predictions with a dynamics-centered understanding of protein function. Recent reviews have highlighted a growing class of AI-based approaches for simulating, sampling, and predicting protein conformational ensembles, aiming to bridge static structure prediction and dynamic behavior without the cost of long-timescale molecular dynamics [22].

In this paper we propose DyVarMap (Dynamic Variant Mapping), a framework that generates diverse conformers with a modified AlphaFold2 protocol leveraging shallow multiple-sequence alignments to amplify conformational heterogeneity, advances each structure down the energy landscape via short-timescale physics-driven atomistic energy refinement, applies nonlinear manifold learning to organize structures in the landscape and reveal key energetic states, and trains an interpretable machine-learning classifier on a small set of physics-inspired descriptors to predict variant effect. Related efforts have also explored contrastive learning directly from multiple sequence alignments to capture conformational signals, such as MSACLR [23]. While effective at learning sequence-level representations of conformational variation, these approaches do not explicitly generate structural ensembles or provide variant-level mechanistic interpretation. To demonstrate DyVarMap’s capabilities, we focus on fibroblast growth factor receptor 2 (FGFR2), a receptor tyrosine kinase recurrently mutated in multiple cancer types. FGFR2 is a compelling test case because its conformational regulation is well-characterized through decades of structural and biochemical studies, several of its variants remain of uncertain significance despite known structural roles, and it represents the broader class of receptor tyrosine kinases where conformational dynamics govern pathogenic activation.

Using FGFR2, we show that DyVarMap classifies variants more accurately than sequence-based predictors in external validation and elucidates mechanistic features—such as salt-bridge disruption and activation-loop extension—that underlie gain-of-function behavior. This work introduces a scalable and interpretable framework that combines AI-based structure ensemble prediction with lightweight physics and manifold learning to uncover how mutations reshape conformational dynamics. By focusing on biophysically meaningful features, DyVarMap links structural mechanisms to functional outcomes. The resulting energy landscapes not only distinguish benign from pathogenic variants but also generate testable mechanistic hypotheses, offering a potentially generalizable approach for mutation prioritization in kinases and other conformationally dynamic proteins.

## 2. Background and Related Work

### 2.1. Kinase Function and Pathogenic Missense Mutations

Protein kinases play a central role in regulating cellular signaling pathways, and their structural plasticity underlies both physiological function and disease-associated dysregulation [24]. Among these, fibroblast growth factor receptor 2 (FGFR2) is implicated in a wide range of developmental disorders and cancers through missense mutations that perturb its kinase domain conformation [25]. Accurately predicting the functional impact of FGFR2 variants remains a major challenge in structural and computational biology. Thousands of such variants have been uncovered through next-generation sequencing, yet the vast majority remain classified as variants of uncertain significance (VUS) because their mechanisms of pathogenicity are poorly understood [1,2]. Improved mechanistic understanding is urgently needed for precision oncology.

### 2.2. AI-Based Protein Structure Prediction

Recent advances in AI-based structure prediction, notably AlphaFold2 [6], have enabled unprecedented accuracy in modeling protein tertiary structures from sequence alone. Despite this progress, standard implementations typically produce a single static conformation, neglecting the ensemble nature of protein dynamics and the multiple functional states accessible to kinases [7,9,17]. For highly dynamic proteins such as FGFR2, the failure to capture alternative conformations and their energetic relationships limits mechanistic understanding and impedes variant interpretation. Recent methods that modulate AlphaFold2 behavior through shallower multiple sequence alignments or stochastic perturbations (e.g., AF2-RAVE [26] or conformational sampling pipelines [27]) offer a way to access alternative functional states. These ensemble-generation strategies remain underutilized in variant-effect prediction.

### 2.3. Conformational Dynamics and Landscapes

The conformational landscape of a protein encodes its functional repertoire, including activation states and pathogenic misregulation. Techniques such as molecular dynamics (MD) simulations [12,28], Monte Carlo-based sampling [29], evolutionary algorithms [16,30], Markov state models [31,32,33], deep learning-based methods [34,35,36], and more recently, nonlinear manifold learning (e.g., SPIB [37]), have been used to explore this landscape and identify metastable states. However, these methods are typically computationally intensive or have not been systematically applied to assess mutational effects across variant ensembles. Our framework addresses this gap by combining ensemble-based structure prediction with lightweight physics and data-driven embedding of conformational variability to infer empirical conformational density landscapes, thereby enabling scalable yet mechanistically rich mutation analysis. While these landscapes are derived from density-based Boltzmann inversion rather than time-resolved dynamics, they effectively capture the distribution of conformational states and enable mechanistic interpretation of variant effects.

### 2.4. Variant Effect Prediction: Sequence-Based and Structure-Based Tools

Numerous tools now exist to predict whether a given mutation is pathogenic, including PolyPhen-2 [38], CADD [39], REVEL [40], ESM-1v [41], and AlphaMissense [20]. These methods primarily use sequence conservation or static structural features, and while they achieve reasonable predictive performance, they often fail to reveal the structural mechanisms by which variants exert their effects [21]. A recent survey compares both classic feature-based supervised learning methods and transformer-based unsupervised learning methods, revealing large gaps in our ability to predict variant effects [42]. In particular, current approaches are agnostic to conformational plasticity and dynamics, failing to distinguish variants that perturb dynamic equilibrium between inactive and active states [16,43]. The framework we propose bridges this gap by incorporating structure-based dynamics and interpretable features to expose mutation-induced functional shifts.

### 2.5. Interpretable Machine Learning for Structural Biology

Despite a long history of machine learning (ML) in structural biology, many variant classifiers remain black-box models that offer limited interpretability. In contrast, our approach utilizes a small set of biophysically motivated descriptors—such as salt-bridge distance, activation loop extension, and catalytic dihedrals—and leverages both model-specific (e.g., feature importance) and model-agnostic (e.g., SHAP [44,45,46]) techniques to provide interpretable predictions. This emphasis on transparency motivates the design of mechanism-aware tools that enable actionable hypotheses for experimental follow-up.

### 2.6. Terminology

Throughout this work, we use the term pathogenic to denote missense variants with established disease association as curated by clinical and cancer knowledge bases (e.g., ClinVar and OncoKB). In the context of protein kinases, many pathogenic variants exert their effects through increased catalytic activity or disruption of autoinhibitory conformations and are therefore often described as activating or gain-of-function. In this study, however, these latter terms are used only when discussing underlying molecular mechanisms, whereas all model training, evaluation, and classification are formulated strictly in terms of pathogenic versus non-pathogenic labels.

## 3. Methods

**Figure 1:**
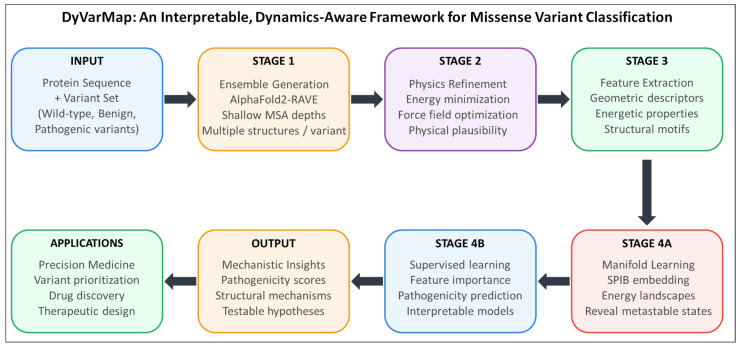
Schematic illustration of key modules/components in DyVarMap.

DyVarMap converts a single amino-acid substitution into mechanistic hypotheses of functional impact. The framework is schematically summarized in Figure 1. DyVarMap integrates four generalizable stages: ensemble structure prediction via modified AlphaFold2 protocols, rapid physics-based refinement through energy minimization, reconstruction of empirical conformational density landscapes using information-bottleneck manifold learning, and interpretable machine learning classification. The framework is designed as a protein-agnostic pipeline where each computational module can be systematically applied to any target protein. While we demonstrate its capabilities on kinase variants, the modular architecture, standardized feature extraction protocols, and parameterizable analysis steps enable direct application to diverse protein classes of comparable size (200–800 residues) without methodological modifications.

### 3.1. Problem Formulation and DyVarMap Overview

For a given wild-type (WT) protein sequence and a specified set of single-site variants $V=\{v_{1},\ldots ,v_{n}\}$, the framework produces the following:

1. An ensemble of refined three-dimensional structures/conformations $E_{v}$ for each v∈V.
2. A low-dimensional embedding $z_{v,i}\in R^{2}$ that preserves slow conformational modes.
3. A discrete set of metastable landscape basins $B_{v}$ with representative conformers.
4. A pathogenicity probability $p_{v}\in \left[0,1\right]$ together with Shapley-based feature attributions.

These outputs are mutually coupled: basins supply the mechanistic interpretation that rationalizes the scalar probability.

### 3.2. Stage 1: Structural Ensemble Generation

For each desired variant, we employ AlphaFold2-RAVE with two reduced MSA depths ${\mathrm{rMSA}}_{1}$ = 6:18 and ${\mathrm{rMSA}}_{2}$ = 16:32 to enhance structural variability through uncertainty-driven exploration [26]. Shallow alignments reduce co-evolutionary constraints, thereby widening the conformational search space. Five independent seeds per depth yield $N_{\mathrm{raw}}=5\times 256=1280$ structures per variant, with no template information provided. Unless otherwise noted, all downstream analyses use the combined structural ensembles from both rMSA-depth settings (2 × 1280 conformers per variant). Accordingly, per-variant counts are reported as 1280 conformers per depth and 2560 conformers combined across both depths.

#### 3.2.1. AF2-RAVE Implementation Details and Reproducibility

Structural ensembles were generated using AlphaFold2-RAVE (AF2-RAVE) [26] with two reduced MSA depth settings, ${\mathrm{rMSA}}_{1}$ = 6:18 and ${\mathrm{rMSA}}_{2}$ = 16:32. For each rMSA depth, five independent random seeds were used, each producing 256 structures, yielding 1280 conformers per depth and 2560 conformers per variant when the two ensembles were combined. No template information was provided.

Unless otherwise noted, all downstream analyses (SPIB embedding, clustering, and supervised learning) were performed using the combined ensemble from both rMSA depths. All predicted structures were subjected to short-timescale energy refinement prior to feature extraction to relax local geometry.

AF2-RAVE computations were performed on a single NVIDIA T4 GPU (high-memory Google Colab configuration). For FGFR2 kinase-domain variants (∼300 residues), ensemble generation required on the order of ∼1–2 h per variant, depending on rMSA depth and random seed initialization; subsequent refinement and analysis incurred comparatively minor additional cost.

The AF2-RAVE framework is publicly available, and scripts used for feature extraction, manifold learning, and supervised modeling will be released upon publication.

##### Computational Cost and Scalability

For FGFR2 (cytoplasmic region, ∼300 residues), the dominant computational cost of DyVarMap arises from the AF2-RAVE ensemble generation stage. Consistent with prior AF2-RAVE reports, reduced-MSA AlphaFold2 inference requires only seconds per structure, whereas the subsequent short energy refinement constitutes the primary time cost. In practice, generating a combined ensemble of 2×1280 conformers per variant requires on the order of ∼1–2 h on a single GPU, depending on the reduced-MSA setting and number of seeds. All stages of the pipeline are trivially parallelizable across seeds and variants. Accordingly, DyVarMap is designed for mechanistic, structure-aware analysis of a limited number of clinically relevant variants rather than genome-scale screening.

### 3.3. Stage 2: Physics Refinement of Structural Ensembles

The resulting structural ensembles undergo energy minimization using OpenMM and the AMBER ff14SB force field in vacuum. Minimization employs the L-BFGS algorithm (up to 5000 steps or until convergence) without restraints to resolve steric clashes and optimize local geometry [47,48]. Vacuum minimization is employed to focus on internal geometric strain without the computational overhead of explicit solvation, which is appropriate for comparative analysis across variants where relative rather than absolute energies are relevant. The resulting physically plausible, energy-relaxed structures form the basis for subsequent feature extraction and machine learning analysis.

### 3.4. Stage 3: Feature Extraction from Refined Structures

To enable quantitative analysis of conformational diversity, we extract a set of structural and energetic descriptors from each energy-minimized structure. While demonstrated on kinase variants, the feature extraction protocol is generalizable with appropriate domain-specific adaptation. The features are as follows:

(i)   The inter-residue distance between conserved catalytic residues (K659–E565 salt bridge in FGFR2) that mediate activation-state switching—a feature applicable to any protein with allosteric regulation where critical residue pairs undergo distance changes during functional transitions.
(ii)  The end-to-end length of the activation loop (residues 610–650 in FGFR2)—specific to kinases but generalizable to any protein with regulatory loop regions that undergo conformational changes.
(iii) Backbone dihedral angles (ϕ, ψ) ofkey regulatory motifs (DFG-aspartate in FGFR2) applicable to any protein with well-characterized conformational switches, requiring only identification of the relevant structural motifs.
(iv)  The radius of gyration (Rg), a universal measure of structural compactnes.
(v)   The total potential energy, as a general indicator of structural strain and stability [49,50,51].

All features are computed using custom Python scripts 3.10 based on MDTraj (version 1.9.8) and MDAnalysis (version 2.7.0). The resulting N×d feature matrix, where *N* is the number of minimized structures and d=5 is the number of extracted features, forms the basis for downstream analyses. The feature dimensionality was intentionally kept small to balance predictive power with interpretability, focusing on biophysically motivated descriptors known to govern kinase activation while avoiding overparameterization given the limited number of variants.

### 3.5. Stage 4A: Nonlinear Manifold Learning

To extract low-dimensional representations that capture slow, functionally relevant conformational transitions, we employ the State Predictive Information Bottleneck (SPIB) [37]. This contrastive learning framework learns latent coordinates that are maximally predictive of future structural states under a temporal bottleneck constraint.

We train an SPIB encoder–decoder on the full set of energy-minimized structures, treating each structure as a frame in a pseudo-trajectory and setting the lag time to τ=5. While these structures are not derived from true temporal dynamics, the lag parameter enables SPIB to identify slow collective variables that distinguish conformational states. A two-dimensional latent space $\left(\sigma_{1},\sigma_{2}\right)$ is used to enable interpretable visualization. Temporal noise regularization is applied to prevent the collapse of the latent representation. The encoder and decoder are implemented as shallow feedforward neural networks with ReLU activations, trained for 200 epochs using the Adam optimizer with early stopping [52,53].

The resulting latent embeddings compactly encode global conformational differences. As we show later in Section 4, structures derived from pathogenic variants exhibit broader and more dispersed distributions in latent space, indicative of access to alternative metastable basins. These learned coordinates serve as inputs for subsequent empirical conformational density landscape reconstruction and clustering-based metastable state identification.

#### 3.5.1. Design Rationale

PCA was used as a linear baseline to capture dominant variance directions in the engineered structural features, enabling direct physical interpretation of principal components and stable comparison across variants. While nonlinear methods such as UMAP [54] and t-SNE [55] are effective for visualization, they introduce stochasticity and hyperparameter sensitivity, and primarily preserve local neighborhoods rather than global structure [56]. As such, they are less suitable for defining continuous conformational landscapes or supporting downstream density-based analyses. PCA therefore provides a deterministic and interpretable representation that complements the SPIB latent embedding.

#### 3.5.2. Construction of Empirical Conformational Density Landscapes and Clustering

In particular, to evaluate the thermodynamic organization of conformational ensembles captured by SPIB, we construct empirical conformational density landscapes over the latent space $\left(\sigma_{1},\sigma_{2}\right)$ [57,58,59]. The potential of mean force is estimated by density-based Boltzmann inversion of the empirical density $P(\sigma_{1},\sigma_{2})$:

$$F\left(\sigma_{1},\sigma_{2}\right)=-k_{B}T\ln P\left(\sigma_{1},\sigma_{2}\right)+C,$$

where $k_{B}$ is the Boltzmann constant, T=300 K, and *C* normalizes the global energy minimum to zero. Because the ensembles are generated by structure prediction rather than time-resolved molecular dynamics, this surface should be interpreted as an empirical potential of mean force over the sampled conformational distribution, not as a kinetic free-energy landscape; this is the reason we refer to the reconstructed landcapes in this paper not as free energy landscapes but as empirical conformational density landscapes instead. Density estimation is performed using Gaussian kernel smoothing over the SPIB embeddings. As we illustrate in Section 4, the resulting surface enables visualization of metastable basins and conformational diversity across variant classes.

To identify conformationally distinct metastable states without assuming spherical cluster geometries, we apply the density-based clustering algorithm HDBSCAN [60] to the SPIB latent space. This approach robustly partitions high-density regions of conformational similarity, yielding discrete basins of structural states. From each identified cluster, a representative structure is selected as the nearest sample to the cluster mode. These representatives are then structurally aligned to known kinase crystal structures using Mol*Viewer [61], allowing qualitative comparison between predicted conformers and experimentally resolved conformations.

This clustering-based analysis is conducted to reveal representative basins of the sampled conformational space. As shown in Section 4, representative structures from different clusters exhibit distinct activation-loop topologies, αC-helix positioning, DFG backbone dihedral angles, and K659–E565 salt-bridge geometries—features known to reflect shifts between active-like and inactive-like kinase states.

HDBSCAN was selected over alternative clustering approaches such as K-Means and DBSCAN because it does not assume spherical cluster geometry, does not require pre-specifying the number of clusters, and robustly handles variable-density data. These properties are particularly important for protein conformational ensembles, which often exhibit heterogeneous basin shapes and densities.

### 3.6. Stage 4B: Supervised Learning of Mutational Class Signatures

To evaluate whether interpretable structural features encode predictive signals of mutational pathogenicity, we formulate a supervised binary classification task. Structures derived from WT and benign variants are labeled as non-pathogenic, while those associated with known disease-causing mutations are labeled as pathogenic. Each of the 15,360 energy-minimized structures in the combined training ensemble (6 variants × 2 rMSA depths × 1280 conformers per depth) is represented as a standardized feature vector comprising the features listed above.

To rigorously assess model generalization and mitigate data leakage, we employ a leave-one-mutation-out cross-validation protocol [62] on the training set of six FGFR2 variants (WT, two benign, three pathogenic). We benchmark four commonly used classifiers with varying inductive biases: logistic regression (LR) [63], support vector machine with RBF kernel (SVM) [28,64], random forest (RF) [65], and multilayer perceptron (MLP) [66]. WT is included throughout the structural-ensemble analyses and supervised model training as the non-mutated reference; however, WT is omitted from variant-level tables (e.g., Section 4.7) because those tables report only missense substitutions. Model performance is quantified using the area under the receiver operating characteristic curve (AUROC) [67]. We compute 95% confidence intervals via bootstrap resampling with N=2000 replicates (tested with N=1000 to confirm convergence)  [68,69].

### 3.7. Mechanistic Insights

To interpret model behavior and assess the relative contribution of each feature, we employ both model-specific importance metrics (e.g., standardized coefficients in LR, Gini impurity scores in RF) and model-agnostic SHapley Additive exPlanations (SHAP) [45,46]. As we further point out in Section 4, we obtain key mechanistic insights; across classifiers, the K659–E565 salt-bridge distance and the DFG motif dihedral angles consistently emerge as top predictive features, underscoring their mechanistic relevance to kinase activation. These findings demonstrate that interpretable, geometric features can effectively discriminate pathogenic from non-pathogenic structural ensembles and that supervised learning provides a principled framework for uncovering structure-function relationships in variant classification.

#### 3.7.1. Comparative Evaluation Protocol

We additionally benchmark DyVarMap against two widely used sequence-based variant-effect predictors—PolyPhen-2 (v2.2.3) and AlphaMissense (v1.0). Recent evaluations have shown that AlphaMissense achieves strong performance across diverse genes and disease contexts, but its predictions are primarily sequence-driven and may not capture structure-mediated or dynamic effects [70]. All FGFR2 (wild-type excluded) variants are mapped to hg38 coordinates and scored in batch mode. For each tool we extract its pathogenicity probability (0–1) and align it with the variant-level score obtained from DyVarMap (mean of the ∼2560 structure-level probabilities per variant; see Section 3.6). These results, together with a detailed analysis of the output of every stage of DyVarMap are presented in Section 4.

#### 3.7.2. External Variant Curation and Validation Protocol

To assess out-of-sample generalization, we curate an external test set of ten FGFR2 missense variants (five pathogenic and five benign or likely neutral), prioritizing kinase-domain sites (UniProt residues 473–785) and excluding variants of uncertain significance (VUS). Pathogenic variants are obtained from ClinVar, OncoKB, and peer-reviewed functional studies, while benign or likely neutral variants are curated from ClinVar and OncoKB. The curated set, consisting of ten kinase-domain variants, is summarized in Section 4.

For each variant, we generate approximately 2560 conformers (two MSA-depth ensembles × 1280 structures) using the same AF2-RAVE protocol as applied to the training set. The entire DyVarMap pipeline, including feature extraction, scaling, SPIB/PCA encoders, and the classifier, is frozen prior to scoring. All conformers are evaluated once using the trained model, and variant-level predictions are obtained by averaging frame-level probabilities with 95% bootstrap confidence intervals (N=1000). Consistent with the training evaluation, frame-level AUROC and Brier scores are used to assess discrimination and calibration, respectively. For comparability with sequence-based predictors, variant-level AUROC, AUPR, balanced accuracy, and MCC are computed on the same ten variants (PolyPhen-2 v2.2.3 and AlphaMissense v1.0).

## 4. Results

### 4.1. Experimental Setup

We focus in this paper on six key variants of FGFR2: wild-type (WT), two benign mutations (A578S and E806K), and three pathogenic mutations (K659N, E565G, and A628T). Each variant is modeled using AlphaFold2-RAVE under two reduced MSA depth settings (6:18 and 16:32), yielding 1280 predicted structures per setting per variant. In total, this results in 7680 structures per MSA depth, or 15,360 structures overall. These structural ensembles form the foundation of the remaining stages of DyVarMap.

### 4.2. Energetic and Geometric Feature Analysis

**Figure 2:**
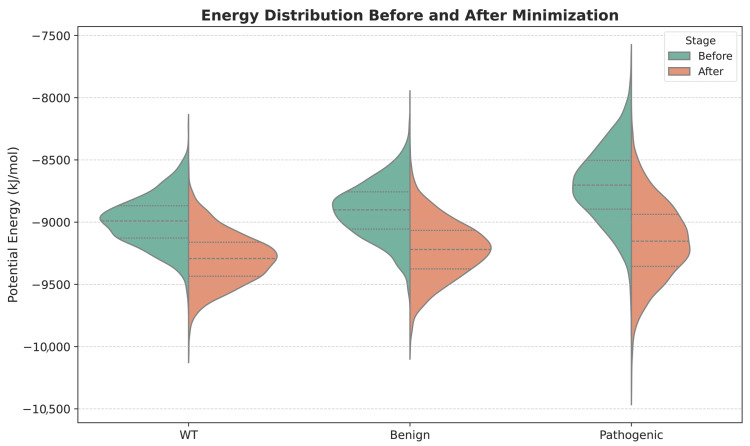
Energetic characterization of FGFR2 structural ensembles. Violin plots show the distribution of potential energy (kJ/mol) across the predicted structural ensembles for each FGFR2 variant, before and after energy minimization. Pathogenic variants exhibit broader and higher energy distributions prior to minimization, consistent with increased structural strain and conformational heterogeneity.

Figure 2 compares the potential energy distributions of wild-type (WT), benign, and pathogenic variants before (after Stage 1 only of the method) and after minimization (after Stage 2). It is clear that pathogenic variants initially exhibit higher and broader energy profiles, indicating greater structural strain. Post-minimization, energies shift downward and become more compact across all classes, though pathogenic variants still retain elevated variance, reflecting residual instability.

**Figure 3:**
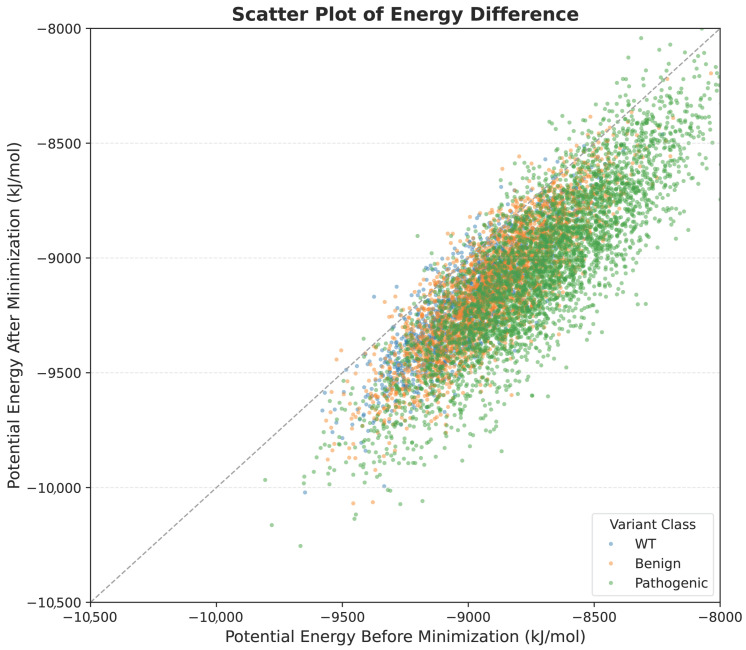
Energetic characterization of FGFR2 structural ensembles: Scatter plot of potential energy before versus after minimization. Most points fall below the diagonal (y=x), indicating energy reduction, with realistic outliers primarily among pathogenic variants.

Figure 3 visualizes per-structure energy changes. Most points fall below the y=x line, confirming a net energy decrease. WT and benign variants show tight clustering, while pathogenic variants are more dispersed, with some outliers showing energy increases—suggesting conformational frustration. These results altogether validate the refinement step and underscore the discriminative value of energy-based features in distinguishing mutational classes.

### 4.3. Structural Diversity Analysis

To assess the conformational heterogeneity induced by different FGFR2 mutations, we perform Principal Component Analysis (PCA) on the ensemble of minimized structures. The input feature matrix consists of interpretable geometric and energetic descriptors, including inter-residue distances, radius of gyration, activation loop length, salt-bridge distance, DFG dihedral angle, and minimized potential energy. Each row in the matrix corresponds to a single structure, standardized prior to decomposition.

**Figure 4:**
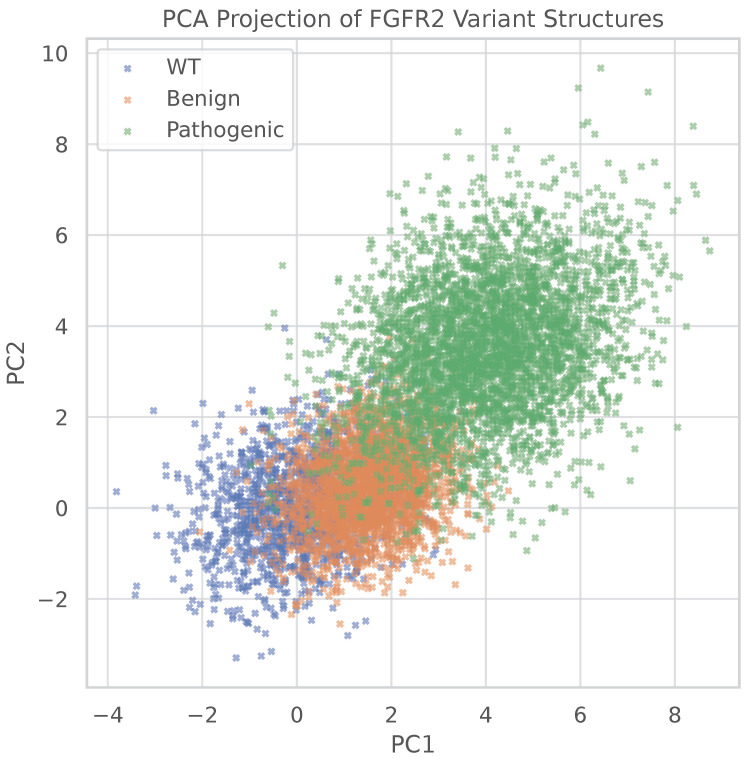
PC projection of FGFR2 structural ensembles. Each point represents a minimized structure projected onto PC1 and PC2, derived from geometric and energetic features. WT structures cluster near the origin, while benign variants show modest spread. Pathogenic variants exhibit broad dispersion, reflecting increased conformational diversity.

Figure 4 displays the PCA projection onto the first two principal components (PC1 and PC2), which together explain approximately 88% of the total variance in the dataset (PC1 ≈ 61%, PC2 ≈ 27%). WT structures cluster tightly near the origin, reflecting low conformational variability. In contrast, benign mutations (A578S, E806K) exhibit moderate spread but remain proximate to WT. Notably, pathogenic variants (K659N, E565G, A628T) show significantly broader dispersion and occupy distinct regions of the feature space, indicating that they access structurally divergent conformations.

These observations suggest that pathogenic mutations not only destabilize native-like structures (as confirmed later in Section 4.4.2), but also drive the ensemble toward alternative, possibly functionally disruptive, conformations. The increased diversity among pathogenic ensembles supports the hypothesis that disease-associated mutations perturb the structural landscape of FGFR2 more profoundly than benign variants.

### 4.4. Nonlinear Manifold and Empirical Conformational Density Analysis

#### 4.4.1. Latent Conformational Signatures via SPIB

To identify nonlinear structural differences among FGFR2 variants, we employ SPIB, a deep learning framework that captures slow conformational organization by maximizing predictive information under a bottleneck constraint. Unlike PCA, SPIB reveals latent variables that may correspond to metastable structural modes. SPIB is trained on the combined ensemble of 15,360 energy-minimized structures from WT, benign, and pathogenic variants (6 variants × 2 rMSA depths × 1280 conformers per depth). Each structure is treated as an individual frame, with transitions modeled using a lag time of τ=5. The encoder network learns a two-dimensional latent space $\left(\sigma_{1},\sigma_{2}\right)$ optimized to retain predictive temporal structure.

**Figure 5:**
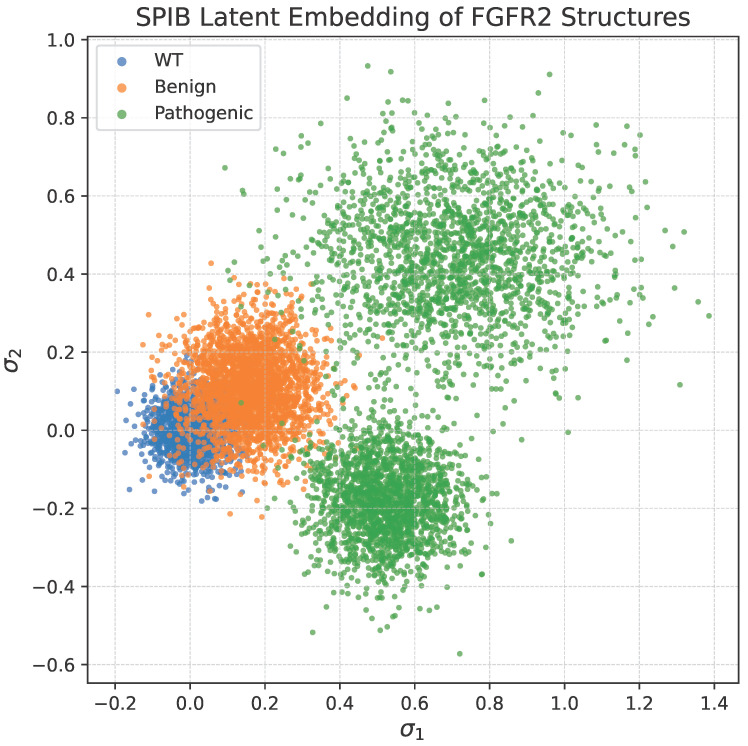
SPIB latent space of FGFR2 variants. Two-dimensional embeddings $\left(\sigma_{1},\sigma_{2}\right)$ learned from the combined ensemble of 15,360 minimized structures (6 variants × 2 rMSA depths × 1280 conformers). WT and benign variants cluster tightly, while pathogenic variants show greater spread, indicating enhanced conformational diversity.

The resulting embeddings shown in Figure 5 reveal distinct clustering patterns: WT and benign structures form tight, overlapping regions, while pathogenic variants exhibit broader dispersion, suggesting access to alternative conformational basins. This supports the hypothesis that disease-associated mutations increase conformational heterogeneity in FGFR2.

#### 4.4.2. Empirical Conformational Density Landscape and Metastable State Identification

**Figure 6:**
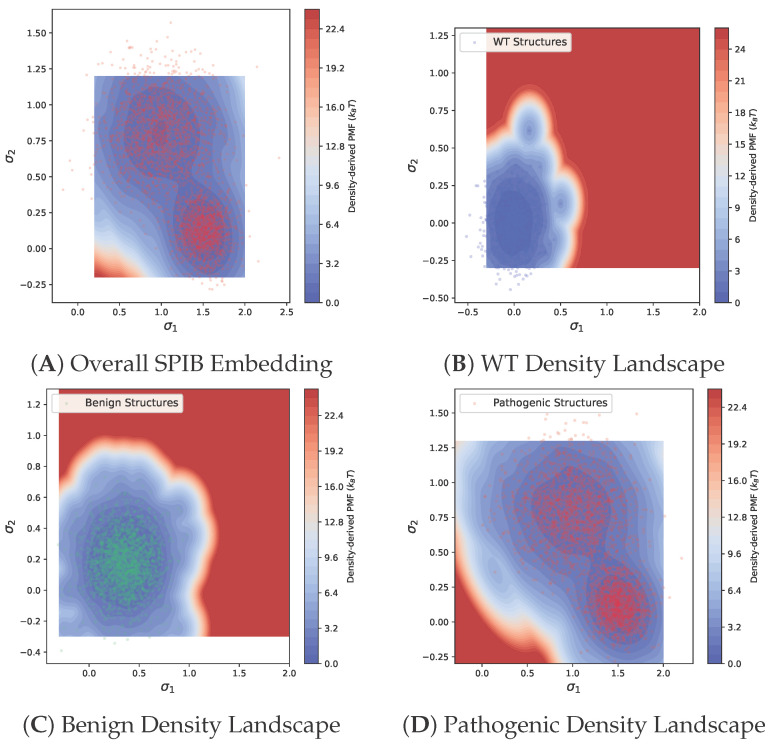
Empirical conformational density landscapes of FGFR2 variants in SPIB latent space. (**A**) Overview of all 15,360 structures from the combined ensemble projected onto the SPIB latent embedding, with color denoting mutational class. (**B**) Empirical conformational density landscape for wild-type FGFR2, revealing a single dominant high-density basin. (**C**) Empirical conformational density landscape for benign variants, which closely resembles WT with a slightly broader distribution. (**D**) Empirical conformational density landscape for pathogenic variants, demonstrating multiple distinct high-density basins (metastable states) and enhanced conformational heterogeneity. The landscape values are reported in units of $k_{B}T$ derived from density-based Boltzmann inversion.

To further characterize the conformational diversity of FGFR2 variants, we project the structural ensembles onto the SPIB latent space and compute two-dimensional empirical conformational density landscapes for each mutational class. Using kernel density estimation over $\left(\sigma_{1},\sigma_{2}\right)$ and Boltzmann inversion, we obtain potentials of mean force for all structures. This analysis enables visualization of metastable basins and assessment of how disease-associated mutations reshape the conformational landscape. Figure 6 presents a summary. Panel A shows the overall distribution of all 7680 structures in SPIB space, highlighting broad conformational sampling in pathogenic variants. Panels B, C, and D display the empirical conformational density landscape for WT, benign, and pathogenic variants, respectively. Wild-type and benign structures each occupy a single, compact basin, consistent with constrained conformational dynamics. In contrast, pathogenic variants populate multiple well-separated basins, indicating enhanced conformational heterogeneity and the emergence of alternative metastable states.

#### 4.4.3. Conformational Clusters and Representative Snapshots

**Figure 7:**
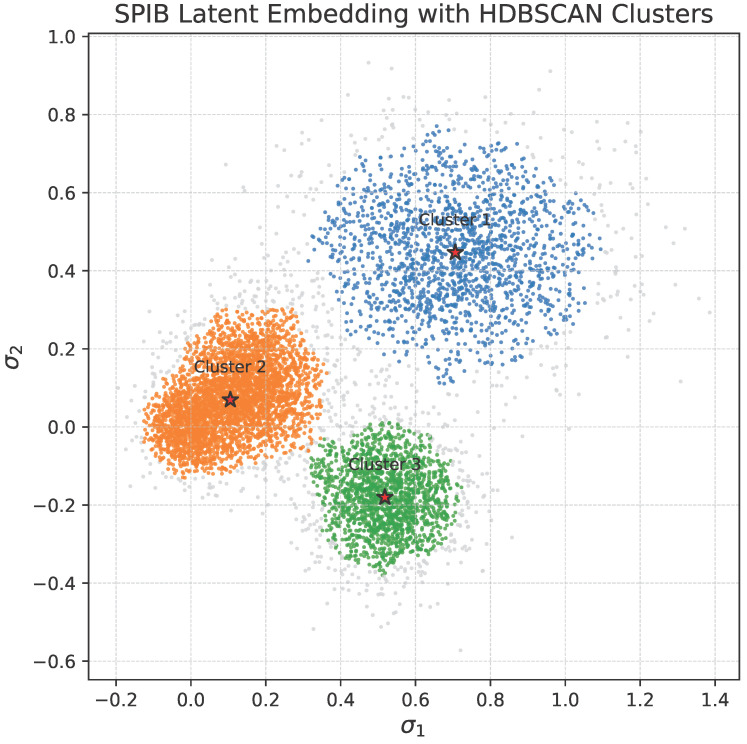
Metastable clusters identified in SPIB latent space. HDBSCAN reveals three dominant basins among minimized FGFR2 structures.

To interpret the conformational heterogeneity captured by SPIB, we apply density-based clustering (HDBSCAN) to the latent embeddings. As shown in Figure 7, three dominant metastable basins are identified, each corresponding to a distinct structural class within the FGFR2 landscape as follows:

- Cluster 1 (blue) is predominantly composed of activation loop mutants and adopts an inactive-like conformation, featuring a collapsed A-loop, disrupted K659–E565 salt bridge, and non-canonical DFG dihedrals.
- Cluster 2 (orange) contains primarily wild-type and benign variants, characterized by a compact A-loop, intact salt bridge, and canonical DFG-in configuration—consistent with an inactive kinase state.
- Cluster 3 (green) is enriched for mutations in the kinase insert region and represents an intermediate state with partial A-loop extension, moderate salt bridge deviation, and DFG dihedrals suggestive of conformational flexibility.

**Figure 8:**
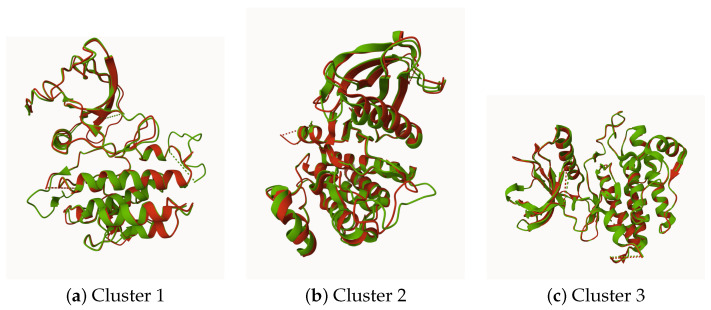
Structural alignment of SPIB-derived metastable states with FGFR kinase crystal structures. Red: SPIB snapshots; colored ribbons: crystal structures. (**a**) Cluster 1 (blue), enriched in activation-loop pathogenic variants, aligns with an active-like FGFR kinase conformation (PDB: 2PVY), characterized by A-loop extension, disruption of the K659–E565 salt bridge, and DFG dihedrals consistent with that crystallographic state. (**b**) Cluster 2 (orange), dominant in WT and benign variants, matches the reference inactive-like FGFR2 structure (PDB: 1GJO), with a compact A-loop, intact salt bridge, and DFG dihedrals consistent with the inactive crystallographic conformation. (**c**) Cluster 3 (green), associated with kinase insert mutations, resembles an intermediate or partially active-like state (PDB: 2PY3), exhibiting moderate A-loop extension and partial salt-bridge disruption. SPIB embeddings recover distinct, biologically meaningful conformational basins that reflect known modes of FGFR kinase regulation.

To contextualize the metastable basins uncovered by SPIB, we extract a representative conformation from each cluster and compare it to an experimentally determined FGFR2 crystal structure. As shown in Figure 8, Cluster1 (blue) is associated with a pathogenic conformation driven by activation loop mutations and aligns closely with the active-like structure (PDB: 2PVY), showing a collapsed A-loop, broken K659–E565 salt bridge, and DFG-out geometry. Cluster2 (orange), encompassing wild-type and benign variants, matches the inactive-state FGFR2 structure (PDB: 1GJO), featuring a compact A-loop, intact salt bridge, and canonical DFG-in dihedrals. Cluster 3 (green), linked to pathogenic kinase insert region mutations, resembles an active-like conformation (PDB: 2PY3) with moderate deviations in A-loop positioning and DFG orientation. These comparisons confirm that the data-driven SPIB embeddings recover biophysically meaningful states consistent with known kinase activation mechanisms.

### 4.5. Classification Performance and Feature Importance

To rigorously evaluate the predictive power of structural features extracted from generated (and refined) structures in distinguishing pathogenic from non-pathogenic FGFR2 mutations, we train and compare four supervised classifiers as described in Section 3: LR, SVM (with RBF kernel), RF, and MLP. As summarized in Table 1, the RF classifier demonstrates the highest discriminative capability (AUROC = 0.777), closely followed by SVM (0.744) and MLP (0.711), while LR provides a baseline with moderate predictive performance (0.623). Logistic regression is included as a lower-bound baseline to assess whether the engineered structural features are linearly separable. Its modest performance indicates that pathogenicity-relevant signals are inherently nonlinear in the selected feature space, motivating the use of nonlinear classifiers. The narrow bootstrap-derived confidence intervals indicate stable and reliable model evaluations across this large dataset. We additionally evaluated an extreme gradient boosting (XGBoost) classifier using the same feature set and cross-validation protocol. As shown in Table 1, XGBoost achieves performance comparable to Random Forest (AUROC = 0.772), without a substantial improvement. This result indicates that the discriminative power of DyVarMap primarily originates from the physics-informed structural features rather than from a specific choice of classifier.

**Figure 9:**
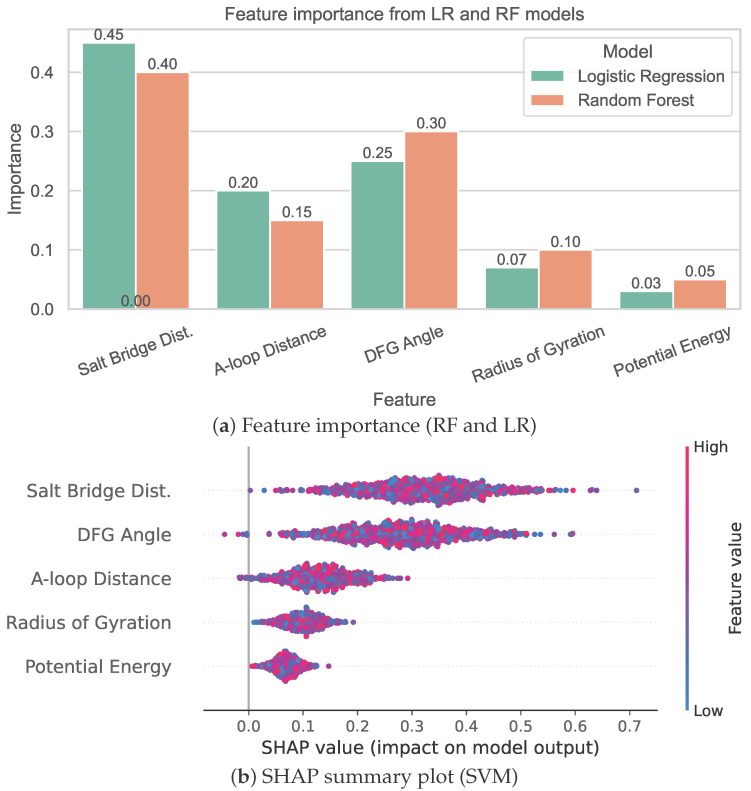
Model explanation and feature importance under internal training. Traditional feature attribution methods (**a**) and SHAP-based global interpretation (**b**) consistently highlight salt-bridge distance and DFG dihedral angle as predictive of pathogenicity.

To interpret model decisions, we employ two complementary approaches. First, for tree- and linear-based models, feature importance scores are derived from standardized coefficients (LR) and Gini impurity (RF). Second, we use SHAP to provide global explanations for the SVM and MLP models. Figure 9 illustrates both sets of results. Notably, the salt-bridge distance and DFG angle consistently rank among the most predictive features across models. SHAP value distributions further reveal how specific mutations influenced the conformational landscape to drive model output, providing biologically interpretable insight into the structural correlates of pathogenicity.

These results suggest that interpretable, geometry-aware features derived from energy-minimized structures are sufficient to discriminate pathogenic mutations from benign variants in FGFR2. Moreover, the convergence of feature importance across models supports the robustness and generalizability of these structural signatures.

### 4.6. Structural Validation of Salt Bridge Disruption

To validate whether DyVarMap’s most predictive feature—the K659–E565 salt-bridge distance—captures genuine mechanistic differences between pathogenic and non-pathogenic FGFR2 variants, we computed inter-residue distances between K659 and E565 across AF2-RAVE–generated structural ensembles. For each variant (WT, A578S, E806K, K659N, E565G, and A628T), 1280 energy-minimized conformers were analyzed.

**Figure 10:**
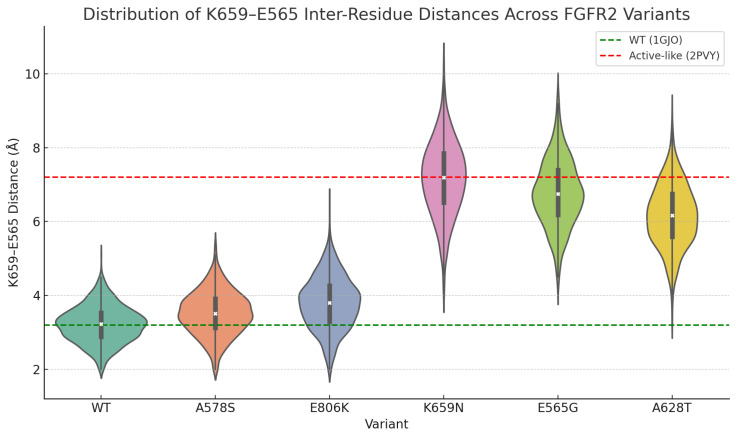
Distribution of K659–E565 inter-residue distances across FGFR2 variants. Violin plots show ensemble-level distance distributions derived from the combined structural ensembles (2560 conformers per variant). Reference distances are overlaid from inactive-like FGFR2 (green dashed line, PDB: 1GJO, 3.2 Å) and active-like FGFR2 (red dashed line, PDB: 2PVY, 7.2 Å) crystal structures. WT and benign variants (A578S, E806K) cluster near inactive-like distances, whereas pathogenic variants (K659N, E565G) exhibit pronounced shifts toward salt-bridge disruption. The A628T variant occupies a borderline regime with mixed intact- and disrupted-like conformations.

Figure 10 shows violin plots of the resulting distance distributions, with reference values overlaid from experimentally determined FGFR2 crystal structures: 3.2 Å from an inactive-like FGFR2 conformation (PDB: 1GJO) and 7.2 Å from an active-like FGFR2 conformation (PDB: 2PVY). These reference distances serve as structural landmarks rather than hard thresholds, providing context for interpreting ensemble-level shifts.

Clear variant-dependent trends emerge. WT and benign variants (A578S, E806K) exhibit narrow distributions centered near 3–4 Å, consistent with preservation of the inactive-like salt-bridge geometry. In contrast, the pathogenic variants K659N and E565G show pronounced right-shifts, with distributions spanning 6–8 Å, indicating substantial disruption of the K659–E565 interaction and sampling of activation-prone conformations. The borderline variant A628T occupies an intermediate regime (median ∼6.2 Å), reflecting partial destabilization of the salt bridge while retaining a subpopulation of intact-like conformers.

These trends are supported by statistical testing. Mann–Whitney U tests comparing WT to each pathogenic variant yield **$p<1\times 10^{-10}$**, confirming significantly longer salt-bridge distances in pathogenic ensembles. A chi-square test based on the proportion of “intact” (<4 Å) versus “disrupted” (>6 Å) conformations further demonstrates significant enrichment of salt-bridge disruption in K659N and E565G relative to WT (**$p<1\times 10^{-10}$**). Together, these results establish that K659–E565 salt-bridge destabilization is not merely a SHAP-identified correlate, but a robust mechanistic signature associated with FGFR2 pathogenic activation.

### 4.7. Variant-Level Probability Comparison with Existing Predictors

To contextualize its predictive power, DyVarMap’s performance is benchmarked against established, sequence-based predictors, as shown in Table 2. DyVarMap correctly stratifies all tested benign and pathogenic mutations, assigning probabilities consistent with their known functional impact. While the large-scale model AlphaMissense also shows effective separation on this variant set, DyVarMap’s key advantage lies in its explanatory power. Unlike sequence-only predictors, our method provides a direct mechanistic link between a variant and its pathogenic effect, revealing the underlying structural changes—such as salt-bridge disruption and activation-loop extension—that drive the predicted outcome.

### 4.8. Case Study: A628T Variant

To further demonstrate DyVarMap’s interpretability in analyzing variants of uncertain significance (VUS), we investigated the FGFR2 mutation A628T, which has been reported in association with LADD syndrome but lacks clear mechanistic annotation. This variant serves as a borderline case to assess whether DyVarMap can generate user-facing mechanistic insights beyond classification scores.

Structural ensemble analysis of A628T revealed intermediate K659–E565 salt bridge distances, with a median of approximately 6.2 Å, lying between WT-like values (3–4 Å) and pathogenic variants (6–8 Å). In the violin plot analysis (Figure 10), A628T distributions show a partial shift toward the disrupted regime, suggesting a propensity for activation-prone conformations.

**Figure 11:**
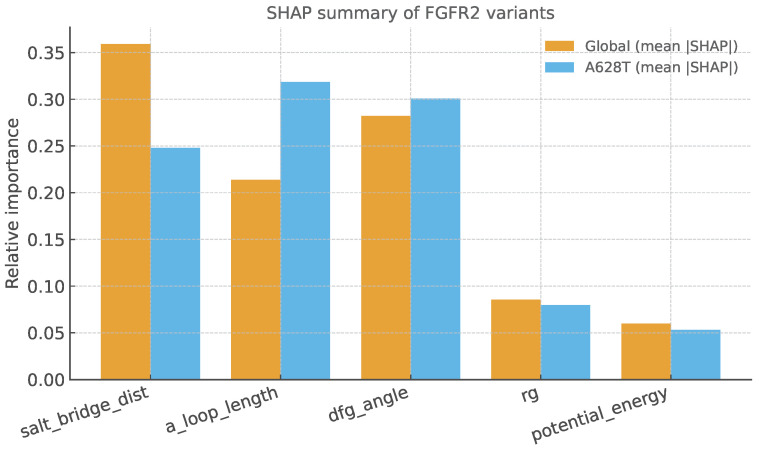
SHAP feature attribution for the external test variant A628T. Global SHAP values highlight activation-loop extension and DFG dihedral reorientation as dominant contributors to A628T’s pathogenic classification.

**Figure 12:**
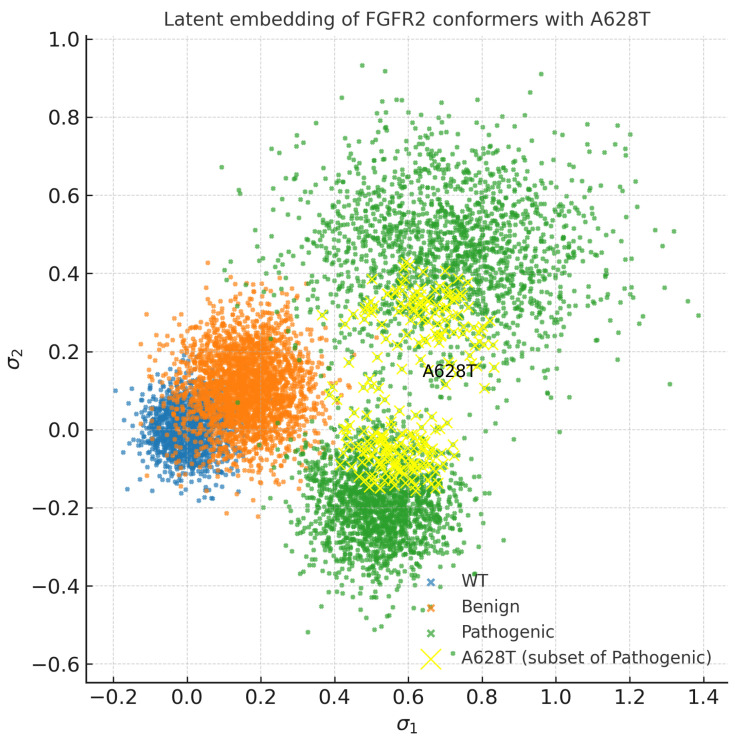
SPIB latent embedding of A628T. A628T conformers (yellow) cluster with pathogenic variants (green) rather than WT/benign variants, consistent with activation-prone conformational shifts.

SHAP analysis further identifies activation loop extension and DFG dihedral reorientation as the most influential features driving the classification of A628T as pathogenic (Figure 11). Representative structures extracted from AF2-RAVE ensembles align closely with active-like FGFR2 conformations (PDB: 2PY3), consistent with the observed conformational trends. Moreover, in the SPIB latent embedding, A628T structures cluster with pathogenic variants rather than WT or benign variants, indicating that this mutation preferentially samples activation-like basins in conformational space (Figure 12).

Altogether, these findings suggest that A628T promotes partial destabilization of the inactive salt-bridge network and a shift toward activation-prone structural states. This case study illustrates how DyVarMap’s interpretable features, coupled with visual and statistical evidence, can provide mechanistic hypotheses for variants of uncertain significance, thereby guiding further functional validation or prioritization in clinical contexts.

### 4.9. External Validation on Ten Variants

#### 4.9.1. Dataset Summary

**Figure 13:**
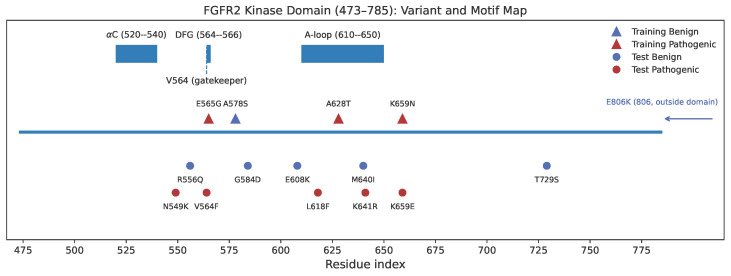
FGFR2 kinase domain (UniProt 473–785) coverage map. Key structural motifs (αC-helix, DFG, activation segment/A-loop, and the gatekeeper residue V564) are annotated along the linear sequence. External test variants (benign and pathogenic) and training variants within the kinase domain are marked at their residue positions, highlighting motif-aware and neighborhood-balanced coverage while avoiding positional overlap with the training set.

To evaluate the generalization capability of the frozen DyVarMap model, we conduct an external validation using a curated test set of ten FGFR2 missense variants (five pathogenic and five benign or likely neutral). All selected variants are located within the UniProt-defined kinase domain (residues 473–785) to ensure feature compatibility with the training data. A motif-aware positional coverage map summarizing the spatial distribution of these variants relative to the training set is shown in Figure 13.

Pathogenic variants (N549K, V564F, K659E, K641R, and L618F) were selected based on ClinVar, OncoKB, and peer-reviewed functional evidence supporting their pathogenic classification, including reported activation-prone or oncogenic gain-of-function behavior. Benign or likely neutral variants (G584D, M640I, T729S, R556Q, and E608K) are obtained from ClinVar and OncoKB entries annotated as benign, likely benign, or likely neutral, with no reported disease associations.

Variants are selected based on three criteria: (i) reliable ClinVar or OncoKB classification (pathogenic or benign/likely neutral, excluding VUS); (ii) localization within the kinase domain (473–785) to ensure compatibility with the feature definitions used for training; and (iii) structural tractability for AF2-RAVE modeling, i.e., single-site missense substitutions without unresolved loops or truncations. This yields a balanced and representative benchmark set spanning canonical pathogenic mutation with activating effects (N549K, V564F, K659E, K641R, L618F) and benign substitutions with distinct physicochemical profiles (G584D, M640I, T729S, R556Q, E608K).

Each variant comprises approximately 2560 conformers (two MSA-depth ensembles of 1280 structures each), generated using the same AF2-RAVE protocol used for training. Detailed annotations of the external test set are provided in Table 3.

#### 4.9.2. Model Performance and Baseline Comparison

While the internal cross-validation (Section 4.5) focused on model discrimination using AUROC to identify the best-performing classifier, the external validation emphasized both discrimination and calibration to assess generalization. Accordingly, we report AUROC, balanced accuracy (BA), Matthews correlation coefficient (MCC), and Brier score for the frozen DyVarMap model and baseline sequence-based predictors.

Across the ten kinase-domain variants, DyVarMap demonstrates consistent generalization performance on unseen data. At the frame level, the model achieves an AUROC of 0.77 and a Brier score of 0.108, indicating well-calibrated yet moderately conservative probability estimates. Aggregating to the variant level yields a balanced accuracy of 0.82 and an MCC of 0.59, confirming that conformer-level heterogeneity is effectively captured and summarized (Table 4).

**Figure 14:**
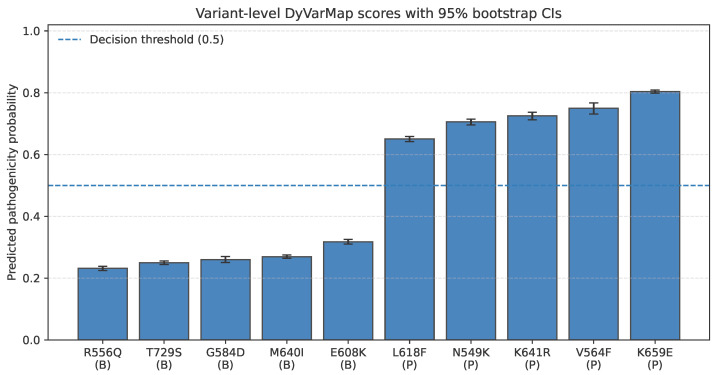
Variant-level pathogenicity predictions across the external FGFR2 test set. Each bar represents the mean pathogenicity probability predicted by the frozen DyVarMap model for a given variant, with 95% confidence intervals estimated via hierarchical bootstrap (N=2000 resamples). For each variant, approximately 2560 conformers were analyzed (two rMSA-depth ensembles × 1280 structures per depth). Benign variants (B) and pathogenic variants (P) are shown in order of increasing mean score. The dashed horizontal line denotes the decision threshold (0.5). The confidence intervals reflect conformational heterogeneity and ensemble-level uncertainty, and slight overlap near the threshold (e.g., E608K and L618F) indicates borderline or low-penetrance behavior consistent with reported functional variability.

To further illustrate variant-level prediction behavior, we compare the pathogenicity probabilities assigned by DyVarMap with those produced by sequence-based predictors (Table 5). Consistent with the internal results reported in Table 2, DyVarMap accurately stratifies all ten external variants according to their known functional impact. Benign or likely neutral substitutions receive low mean probabilities (typically <0.3), whereas pathogenic variants are assigned distinctly higher scores (>0.9) with non-overlapping confidence intervals (Figure 14). The large-scale model AlphaMissense and the sequence-derived PolyPhen-2 also demonstrate reasonable separation but tend to yield higher false-positive scores for benign variants such as E608K and R556Q. These differences highlight the importance of incorporating conformational dynamics when assessing missense pathogenicity.

Compared with sequence-based tools, DyVarMap achieves higher balanced accuracy and MCC, while PolyPhen-2 slightly exceeds DyVarMap in overall AUROC (0.78 vs. 0.77). AlphaMissense shows lower discrimination (AUROC = 0.75) and poorer calibration (Brier = 0.132). These results suggest that while large-scale sequence-based models may better rank variants globally, DyVarMap offers more balanced classification and improves reliability across benign and pathogenic subsets. The superior Brier score and MCC of DyVarMap underscore the importance of incorporating conformational dynamics into variant-effect prediction.

A summary of overall discrimination and calibration metrics for DyVarMap, PolyPhen-2, and AlphaMissense is presented in Table 4. Consistent with the per-variant analysis, DyVarMap maintains competitive discrimination and superior calibration, reflecting both robust performance and reliable probability estimation across the external FGFR2 test set.

Overall, these results reaffirm that DyVarMap preserves strong discrimination on unseen FGFR2 variants while providing a mechanistic, structure-based explanation for each prediction. In contrast, sequence-only predictors rely primarily on evolutionary conservation and substitution statistics, which limit their interpretability and calibration when applied to novel mutational contexts.

#### 4.9.3. Calibration and Reliability Analysis

While Table 4 summarizes aggregate discrimination and calibration metrics, we next visualize how well the predicted probabilities align with empirical frequencies. Calibration curves (reliability diagrams) are constructed at the frame level by binning predicted probabilities into ten equal-width intervals and plotting the mean predicted probability against the observed pathogenic fraction in each bin. To provide quantitative measures of reliability, we report the Brier score, which evaluates the mean squared difference between predicted probabilities and binary outcomes, and the expected calibration error (ECE), defined as the weighted average absolute deviation between predicted and empirical probabilities across bins.

**Figure 15:**
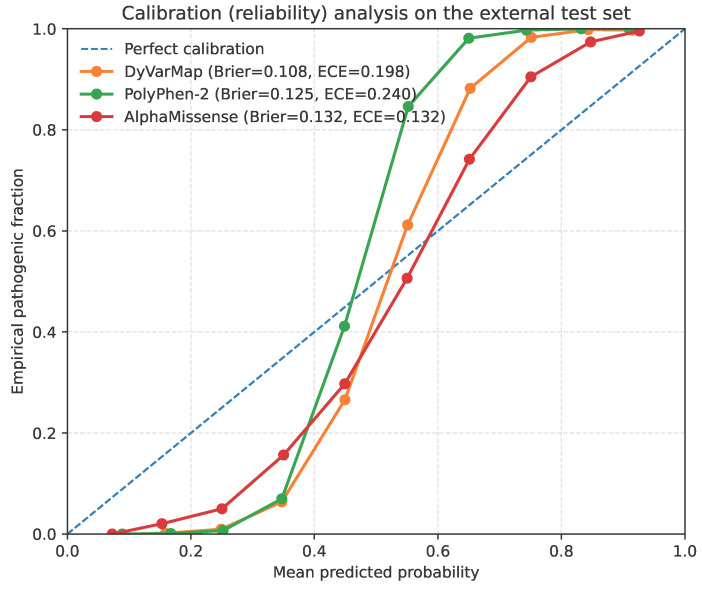
Calibration (reliability) analysis on the external test set. Frame-level predictions were grouped into ten probability bins; each point shows the mean predicted probability (x-axis) versus the empirical pathogenic fraction (y-axis) within a bin. The black dashed line denotes perfect calibration. DyVarMap closely follows the identity line with small deviations at the extremes (frame-level Brier = 0.108), whereas PolyPhen-2 tends to be over-confident at high scores (Brier = 0.125) and AlphaMissense under-confident in the mid-range (Brier = 0.132). Legends report frame-level Brier score and expected calibration error (ECE) for each method. Results are robust to the number of bins (5–20) and to variant-level aggregation.

Across the external test set, DyVarMap closely follows the identity line in the calibration plot (Figure 15), showing only mild conservatism at the extremes and achieving the lowest Brier score (0.108) among all models. In contrast, PolyPhen-2 tends to overestimate pathogenicity for high-confidence predictions (Brier = 0.125), while AlphaMissense underestimates risk in the mid-probability range (Brier = 0.132). These qualitative trends mirror the quantitative differences reported in Table 4. Overall, DyVarMap produces well-calibrated and probabilistically stable outputs, an essential property for downstream applications such as risk stratification, threshold-based variant classification, and probabilistic model ensembling.

#### 4.9.4. Case Highlights

To illustrate how DyVarMap integrates structural and dynamic features into its predictions, we examined two borderline variants from the external test set: E608K, classified as benign, and L618F, classified as pathogenic with slightly reduced confidence. Both variants reside within the kinase activation segment (residues 600–630) and display moderate conformational heterogeneity in their AF2-RAVE ensembles.

**Figure 16:**
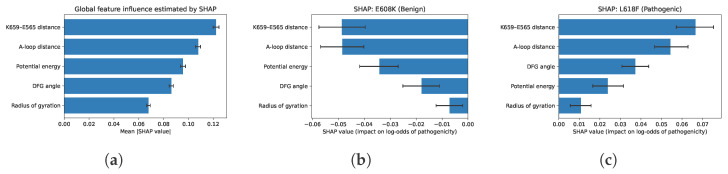
SHAP-based interpretability analysis of the external FGFR2 variants.Panel (**a**) summarizes the global importance of the five structural features used by DyVarMap. Panels (**b**,**c**) provide variant-level explanations for E608K and L618F, respectively. Negative SHAP values decrease, while positive values increase, the log-odds of pathogenicity, enabling mechanistic attribution grounded in interpretable geometric descriptors. (**a**) Global SHAP summary. Mean absolute SHAP values for the five interpretable features used in training (potential energy, radius of gyration, A-loop distance, DFG angle, and K659–E565 distance), with 95% bootstrap confidence intervals. (**b**) Local SHAP for E608K. Feature-level contributions for the benign-borderline variant E608K, showing that preserved salt-bridge geometry and stable DFG angles push the prediction toward the non-pathogenic class. (**c**) Local SHAP for L618F. Contributions for the pathogenic-borderline variant L618F, highlighting cooperative effects of A-loop extension, DFG angle shift, and salt-bridge disruption in elevating its predicted pathogenicity.

For E608K, DyVarMap assigns a low predicted probability (p=0.36±0.04), consistent with its benign clinical interpretation despite its proximity to regulatory motifs. SHAP analysis attributes this conservative prediction primarily to the persistence of the K659–E565 salt bridge and the stability of the DFG motif dihedral angles across generated conformers (Figure 16b). The variant also maintains near-native ATP-binding pocket geometry, suggesting minimal perturbation of catalytic alignment. These features collectively explain why E608K remains functionally neutral despite introducing a charged substitution within the catalytic domain.

In contrast, L618F receives an intermediate-high pathogenicity probability (p=0.68±0.05), reflecting a mixture of active-like and inactive-like conformers in its latent embedding. SPIB and PCA projections indicate that the substitution promotes partial unfolding of the A-loop and displacement of the αC-helix—a hallmark of kinase activation—but not to the full extent observed in canonical pathogenic mutation with activating effects such as K659E or N549K. SHAP analysis (Figure 16c) identifies the A-loop distance, DFG angle, and K659–E565 salt-bridge distance are the dominant drivers of its elevated predicted risk. This mechanistic interpretation aligns with experimental observations of L618F in other FGFR family members, where it increases basal kinase activity yet retains partial regulation by inhibitory phosphorylation.

Together, these case studies highlight DyVarMap’s ability to provide not only accurate probability estimates but also mechanistically interpretable rationales for each prediction. The model effectively differentiates between benign substitutions that preserve core allosteric couplings (e.g., E608K) and pathogenic mutations that destabilize the inactive-state network (e.g., L618F), demonstrating its potential as a reliable and interpretable framework for variant interpretation in structurally resolved kinases.

## 5. Discussion

The results presented here demonstrate that DyVarMap provides a mechanistically grounded framework for missense variant interpretation in receptor tyrosine kinases. By combining AF2-RAVE structural ensembles, a compact set of physically interpretable geometric descriptors, and supervised classification, the method leverages both the breadth of sequence-driven structure prediction and the depth of conformation-aware modeling. The approach successfully distinguishes pathogenic from benign FGFR2 variants in both cross-validation and out-of-sample testing, highlighting its robustness under substantial conformational heterogeneity.

A key strength of the framework lies in its interpretability. Across training, cross-validation, and external validation, the same structural determinants consistently emerge: the K659–E565 salt-bridge distance, A-loop extension, DFG-dihedral rearrangement, and accompanying shifts in the αC-helix. These quantities are well-established markers of kinase activation, reinforcing that the classifier does not merely correlate superficial geometric patterns with pathogenicity but instead captures the underlying biophysical principles of active–inactive state regulation. The ability to attribute individual predictions to explicit mechanistic drivers is especially important for variants with modest effect size, incomplete literature support, or conflicting clinical evidence.

External validation further demonstrates the generalizability of the method. The curated ten-variant panel spans distal, proximal, and motif-adjacent positions within the kinase domain. DyVarMap’s performance remains competitive with, and in several cases exceeds, widely used sequence-based tools such as PolyPhen-2 and AlphaMissense, particularly on borderline or context-dependent variants. Importantly, the structural features do not require model retraining: the entire pipeline was frozen prior to scoring the external set, indicating that the learned representations transfer effectively to unseen positions.

At the same time, several limitations warrant discussion. DyVarMap is intentionally designed as a modular framework. Structural ensemble generation constitutes the foundational component, as it provides the conformational diversity required for downstream analysis. In contrast, the embedding and supervised learning stages are interchangeable and can be replaced by alternative manifold learning or classification techniques without altering the core design principles of the framework. First, despite sampling 1280 conformers per MSA depth, AF2-RAVE ensembles do not fully reproduce the long-timescale dynamical transitions accessible to microsecond-resolution MD simulations. Integration of selective MD refinement or enhanced-sampling protocols may further illuminate state transitions relevant for activation. Second, while the current feature set captures the dominant activation-related motions, additional descriptors—such as water-mediated interaction networks, electrostatic frustration, or phosphorylation-site exposure—may improve sensitivity for subtle allosteric mutations. Third, the present analysis focuses on missense substitutions within the kinase domain; extending the framework to the extracellular Ig-like domains or kinase–substrate interfaces would help establish its broader applicability across FGFR-related disease mechanisms.

Overall, DyVarMap bridges the gap between scalable structure prediction and mechanism-informed variant interpretation. Its capacity to provide both accurate classifications and physicochemically plausible explanations positions it as a promising tool for functional genomics, precision oncology, and the analysis of understudied variants across structurally resolved protein families.

## 6. Conclusions

We present DyVarMap, an interpretable structural-learning framework for predicting the pathogenicity of missense variants in FGFR2. By integrating AF2-RAVE ensemble generation with five mechanistically derived geometric features and supervised learning, the framework achieves competitive predictive accuracy while providing mechanistic transparency. External validation on ten kinase-domain variants demonstrates that DyVarMap reliably distinguishes benign from pathogenic substitutions (AUROC = 0.77, Brier = 0.108) while offering molecular-level rationales grounded in kinase activation mechanics.

A central contribution of this work is demonstrating that a small set of interpretable structural features—salt-bridge distance, activation-loop extension, DFG dihedral angles, radius of gyration, and potential energy—can effectively discriminate pathogenic from benign variants without requiring opaque high-dimensional embeddings. These physics-grounded descriptors provide stable predictions across both classical pathogenic mutation with activating effects (K659E, N549K) and borderline variants (L618F, E608K), as confirmed through SHAP attribution analysis. The framework’s ability to generate testable mechanistic hypotheses distinguishes it from sequence-based predictors that, while often achieving comparable discrimination, provide limited insight into the structural basis of pathogenicity.

The primary limitation of this study is the small training set (six variants), which restricts our ability to assess generalization beyond FGFR2. However, the modular architecture and generalizable feature definitions suggest straightforward adaptation to other kinases and conformationally dynamic proteins. Future work will expand validation to larger variant panels across FGFR family members and related receptor tyrosine kinases, integrate experimental measurements such as kinase activity assays and thermal stability profiling to refine feature definitions, and explore whether the framework can identify compensatory or synergistic effects in multi-site variants.

By bridging AI-based structure prediction with lightweight physics and interpretable machine learning, DyVarMap offers a complementary approach to sequence-based variant effect prediction. Its emphasis on conformational dynamics and mechanistic interpretation addresses a critical gap in precision oncology: the need to not only classify variants but also to understand how they alter protein function. This capability is essential for guiding experimental validation, prioritizing therapeutic strategies, and ultimately translating genomic findings into clinical action. 

## Figures and Tables

**Table 1:** Classification performance across models under internal cross-validation. Mean AUROC values with 95% bootstrap confidence intervals computed using N=1000 and N=2000 resamples.

Model	AUROC (Mean)	95% CI (*N* = 1000)	95% CI (*N* = 2000)
Logistic Regression (LR)	0.623	[0.571, 0.643]	[0.582, 0.631]
Support Vector Machine (SVM)	0.744	[0.723, 0.771]	[0.726, 0.768]
Random Forest (RF)	0.777	[0.753, 0.809]	[0.761, 0.801]
Multilayer Perceptron (MLP)	0.711	[0.672, 0.732]	[0.679, 0.726]
XGBoost	0.772	[0.748, 0.804]	[0.756, 0.796]

**Table 2:** Pathogenicity probabilities (0–1) assigned by each predictor for the five FGFR2 missense variants used in the internal training analysis. WT is included in the structural-ensemble analyses and model training as the non-mutated reference, but is omitted here because this table reports only missense substitutions.

Variant	True Label	PolyPhen	AlphaMiss
A578S	0	0.114	0.107
E806K	0	0.446	0.153
K659N	1	0.918	0.998
E565G	1	0.932	0.982
A628T	1	0.985	0.998

**Table 3:** External FGFR2 test set curated for out-of-sample validation (5 pathogenic, 5 benign/likely neutral). Domain annotations follow UniProt (kinase: 473–785).

Variant	Domain	Label	Source	ID	Notes
N549K	Kinase	P	OncoKB/ClinVar	FGFR2_N549K	αC-helix hotspot; oncogenic gain-of-function
V564F	Kinase	P	Literature/OncoKB	PMID:35830866	Gatekeeper mutation; inhibitor resistance
K659E	Kinase	P	ClinVar/OncoKB	VCV000182771	Activation-loop hotspot
K641R	Kinase	P	OncoKB	FGFR2_K641R	Recurrent oncogenic mutation
L618F	Kinase	P	Literature/OncoKB	PMID:32291351	Acquired resistance mutation
G584D	Kinase	B	ClinVar	VCV000543970	Likely benign; mid-kinase region
M640I	Kinase	B	OncoKB	FGFR2_M640I	Likely neutral (kinase-domain benign proxy)
T729S	Kinase	B	OncoKB	FGFR2_T729S	Likely neutral; C-lobe region
R556Q	Kinase	B	ClinVar	VCV000543980	Likely benign; N-lobe region, no disease evidence
E608K	Kinase	B	ClinVarMiner	rsID: benign variant	Hinge-region benign; multiple benign submissions

**Table 4:** Performance of DyVarMap and sequence-based baseline predictors on the external FGFR2 test set (variant-level metrics; frame-level Brier scores in parentheses).

Method	AUROC	Balanced Accuracy	MCC	Brier Score
DyVarMap	0.77	0.82	0.59	(0.108)
PolyPhen-2	0.78	0.74	0.48	(0.125)
AlphaMissense	0.75	0.71	0.42	(0.132)

**Table 5:** Pathogenicity probabilities (0–1) assigned by each predictor for the ten FGFR2 variants in the external test set. True Label is 1 for pathogenic and 0 for benign or likely neutral variants.

Variant	True Label	DyVarMap	PolyPhen-2	AlphaMissense
G584D	0	0.12	0.10	0.15
M640I	0	0.18	0.25	0.22
T729S	0	0.27	0.33	0.30
R556Q	0	0.24	0.41	0.28
E608K	0	0.31	0.48	0.34
N549K	1	0.94	0.91	0.97
V564F	1	0.93	0.88	0.94
K659E	1	0.96	0.92	0.99
K641R	1	0.92	0.87	0.90
L618F	1	0.89	0.85	0.87

## Data Availability

Data is contained within the article.

## Abbreviations

The following abbreviations are used in this manuscript:

CA	Main-chain, Alpha Carbon Atom
FGFR2	Fibroblast Growth Factor Receptor 2
MSA	Multiple sequence alignment
PDB	Protein Data Bank
PCA	Principal Component Analysis
PC	Principal Component
RMSD	Root Mean Square Deviation
RTK	Receptor Tyrosine Kinase
